# Congenital Aplasia of the Common Carotid Artery: A Comprehensive Review

**DOI:** 10.1155/2019/9896138

**Published:** 2019-12-23

**Authors:** L. Vasović, M. Trandafilović, S. Vlajković

**Affiliations:** ^1^Faculty of Medicine, University of Niš, 81 Blvd. Dr. Zoran Djindjić, Niš 18000, Serbia; ^2^Department of Anatomy, Faculty of Medicine, University of Niš, 81 Blvd. Dr. Zoran Djindjić, Niš 18000, Serbia

## Abstract

In an attempt to describe the morphofunctional consequences of uni- and bilateral aplasia of the common carotid artery (CCA), which is usually a vascular source of the external carotid (ECA) and internal carotid (ICA) arteries, we investigated online databases of anatomical and clinical papers published from the 18th century to the present day. We found 87 recorded cases of uni- and bilateral CCA aplasia in subjects from the first hours to the eighth decade of life, which had been discovered in 14 (known) countries. Four crucial parameters were described: the embryology of the carotid arteries, morphophysiology of the carotid arteries, CCA aplasia, and unilateral versus bilateral CCA aplasia, including history, general data, diagnosing, vascular sources, caliber, course of the separated ECA and ICA, associated vascular variants, and pathological disorders. To complete the knowledge of the morphofunctional consequences of the absence of some artery of the carotid system, and risking the possibility of repeating some words, as “carotid artery”, or “carotid aplasia” and the headings from our previous article about bilateral ICA absence, this review is the first in the literature that recorded all cases of the CCA aplasia published and/or cited for the past 233 years. Main characteristic of the CCA absence is its association with 21 different diseases, among which the aneurysms were in 13.69% of cases, and 17.80% of cases were without pathology.

## 1. Introduction

### 1.1. Embryology of the Carotid Arteries

The data about human arteries confirm their initial development on the 19th day of gestation [1]. A pair of longitudinally channels arising in a paramedian location of the embryo becomes paired dorsal aortae (DAs). From 21 to 25 days, the heart tubes fuse into a primitive heart, and the ventral aortic sac stays bilaterally connected to the DAs by the first primitive aortic arch (PAA). Although by 32 days, six pairs of PAAs are formed, the first and second PAAs already disappear at day 29 of gestation. By 6 weeks [2], or at an embryo of 12–14 mm [3], a regression of DA occurs between the third and fourth PAAs.

Pansky [4] described that a short portion of the right primitive ventral aorta between the fourth and sixth PAAs forms the brachiocephalic trunk (BT) and a part of the aortic arch, while Jerius et al. [5] noted that a common origin of the right third and fourth PAAs elongates with growth of the embryo, forming the BT. The proximal part of the right fourth PAA persists as the right subclavian artery (SA) up to the origin of the internal thoracic artery, while the distal part of the right fourth PAA gets regressed. The distal part of the left fourth PAA also regresses and its proximal part forms a small segment of adult aortic arch between the origin of the left common carotid artery (CCA) and left SA [3]. Since the left CCA usually starts directly from the arch of the aorta, which develops from the left fourth PAA, it can be said that this PAA also stands as a precursor of the left CCA [2]. The right CCA arises from the BT, which is a remnant of the ventral aortic sac. New branches from the ventral aspects of the right and left third PAAs form the right and left external carotid arteries (ECAs) which, in some instances, may include portions of the first and second arch arteries [6]. The third PAA becomes the carotid sinus, and with the cranial DA forms the internal carotid artery (ICA), while the cranial ventral aorta becomes the ECA [7]. However, the root of the ECA can originate only from the proximal portion of the third PAA, which normally becomes the CCA [3].

### 1.2. Histophysiology of Embryonal Precursors of Carotid Vessels

The breaking of the dorsal aorta between the third and fourth PAAs, called the ductus caroticus (DC), according to Congdon [8], as cited [9], occurs because the third PAA is directed cranially, whereas the fourth PAA is directed caudally. This creates a stagnant area between the points of attachment of the two PAAs, which is ruptured by the tension caused by caudal descent of the heart. Regression of the DC on both sides induces a connection of the ventral aortic sac through the third PAA to the cranial extension of the DA. The involution of both dorsal aortae between the third and fourth PAAs seems to be due to vasoconstriction at these two sites, related to a decrease in blood flow through these short segments [10]. The flow in the third PAAs is directed cranially into the rostral portion of the dorsal aortae, while the current in the fourth PAAs is directed into the caudal dorsal aortae. The contracted segments of the dorsal aortae become progressively thinner, until they completely disappear, allowing the third PAAs and the rostral dorsal aortae to further develop into portions of the carotid arteries.

## 2. Morphophysiology of Definitive Carotid Arteries

Bilaterally, the CCA and its terminal branches—ECA and ICA are major blood vessels that supply the structures of the head and neck. However, the waveforms in the ECA and ICA are typical, because their resistive indices are determined by the corresponding regions and organs supplied by them, without an influence of the CCA on the nature of the ICA and ECA waveforms [11]. Both distensibility and cross-sectional compliance of the CCA decreased linearly with age, starting in the third age decade [12].

The right CCA, as an ascending branch of the brachiocephalic trunk (BT), averaged 9.4 cm in length, while the left CCA, as a direct branch of the arch of the aorta averaged 13.4–14.4 cm; the caliber of the both CCAs accounted about 8 mm [2]. Usually, there are no side branches on their course; however, some branches of the ECA or other arteries can branch off from the CCA, uni- or bilaterally [10].

## 3. CCA Aplasia

A research of the cause of the CCA absence was the topic of many works over the centuries.

Macalister in 1886 [13] has noted that a persistence of the DC and an obliteration of the transverse part of the third PAA cause the ICA to arise independently from the aorta. This opinion was supported 50 years later by the facts that no carotid sinus nor sinus nerve were presented, which are closely connected in the normal development of the third PAA [14].

If the DC persists, while the fourth PAA involutes, the third PAA could become the definitive arch of the aorta with separate ICA and ECA arising from it; when the anomaly is present on the right side, the ECA arises from the BT, whereas the ICA arises from the SA [5, 14−18], or both ECA and ICA could have separate origins from the BT [7, 19–29], or from the right SA [3].

Lie [30], as cited [5], described three theories about the separate origins of the ECA and ICA. His first theory is based on works of embryologists at beginning of the 20th century, which have described that an involution of the entire third PAA of affected side is accompanied by the persistence of DC. The second theory is based on the works of embryologists of his time, which noted that a failure of usual migration of the ECA and ICA toward each other to create a CCA would result in their separate origins. The third theory postulated the failure of DC to involute, causing the disappearance of a distal portion of the third PAA; the proximal portion of the third PAA, instead of comprising the CCA, now becomes the proximal portion of the ECA. Lie [30] also described, as cited [31], that the ventral pharyngeal artery (VPA) sometimes arises from the third PAA directly at the location of the carotid bifurcation; however, in the condition of agenesis of the third PAA, the VPA will not sprout out, leading to the absence of CCA and ECA.

### 3.1. Aplasia of the Left CCA

**Figure 1:**
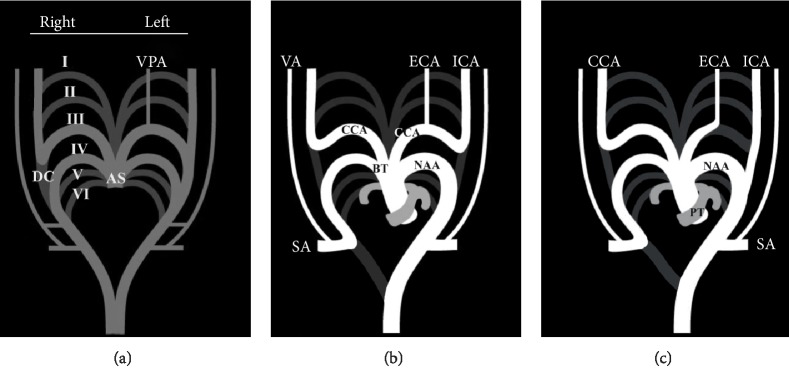
Modified schemes [10] of the normal development of all carotid arteries (a–b), and separate origin of the left external and internal carotid arteries (c). I–VI, primitive aortic arches; DC, ductus caroticus; VPA, ventral pharyngeal artery; NAA, normal aortic arch; BT, brachiocephalic trunk; CCA common carotid artery; ICA, internal carotid artery; ECA, external carotid artery; VA, vertebral artery; SA, subclavian artery; PT, pulmonary trunk.

It was noted that the separated ECA in the monkey is formed by the sequent ventral aortic root of the cranial part from the fourth PAA on the left side, while the left ICA is formed by the sequent dorsal aortic root of the cranial part from the left fourth PAA; consequently, the left CCA is not formed [32]. Hovewer, it was noted, too, that the absence of the left CCA in a human is a consequence of the persistence of the left DC with an involution of the left third PAA (Figure 1); the left ICA would thus originate from the left fourth PAA proximal to the origin of the left SA [1]. In addition, the left ECA could originate either from a remnant of the third PAA or directly from the left fourth PAA [10]; in either case, the adult phenotype would produce a left ECA originating between the BT and the left ICA.

The persistence of the entire third PAA on one side and the involution of the contralateral DC with the fourth PAA on both sides can result in the cervical aortic arch (CAA) with separate origins of the ECA and ICA from it [33, 34]. Moncada et al. [35] have described three theories of separate origins of the ECA and ICA stemming from the CAA; the first theory was based on the failure of the****descent of the otherwise normal fourth PAA; the second theory was based on the incorporation of the third or second PAAs into the adult aortic arch, and involving of the second or first PAA into proximal part of the ICA; and the third theory was based on the fusion of the third and fourth PAAs on the involved side.

### 3.2. Aplasia of the Right CCA

**Figure 2:**
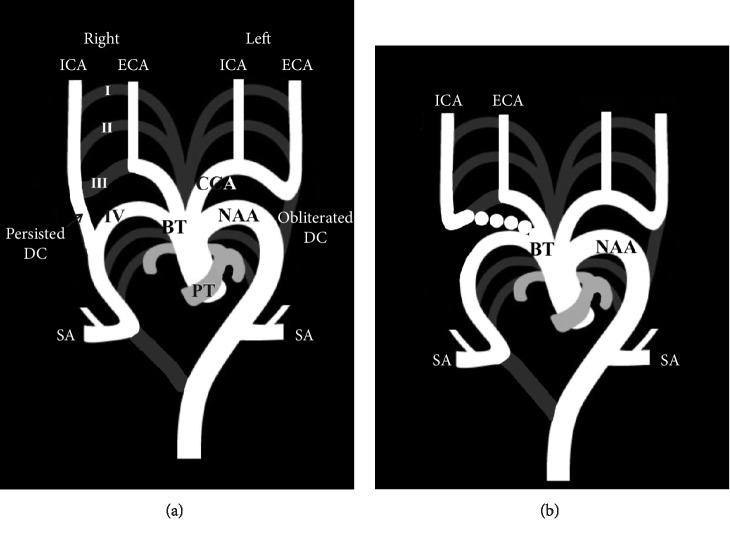
Modified schemes [10] of the separated origin of the right external carotid (ECA) and internal carotid (ICA) arteries from the brachiocephalic trunk (BT) and right subclavian artery (SA) in the first scheme (a), and from the BT in the second scheme (b). I–VI, the first four (from six) primitive aortic arches; DC, ductus caroticus; NAA, normal aortic arch; CCA, common carotid artery; BT, brachiocephalic trunk; PT, pulmonary trunk; SA, subclavian artery.

In the greatest number of cases, the separated right ECA and ICA originated from the BT and right SA, respectively, or only from the BT (Figure 2). Maybody et al. [21] explained a proximal origin of the right ICA from the BT in relation to the right ECA by an extra twisting of the separate ICA and ECA.

**Figure 3:**
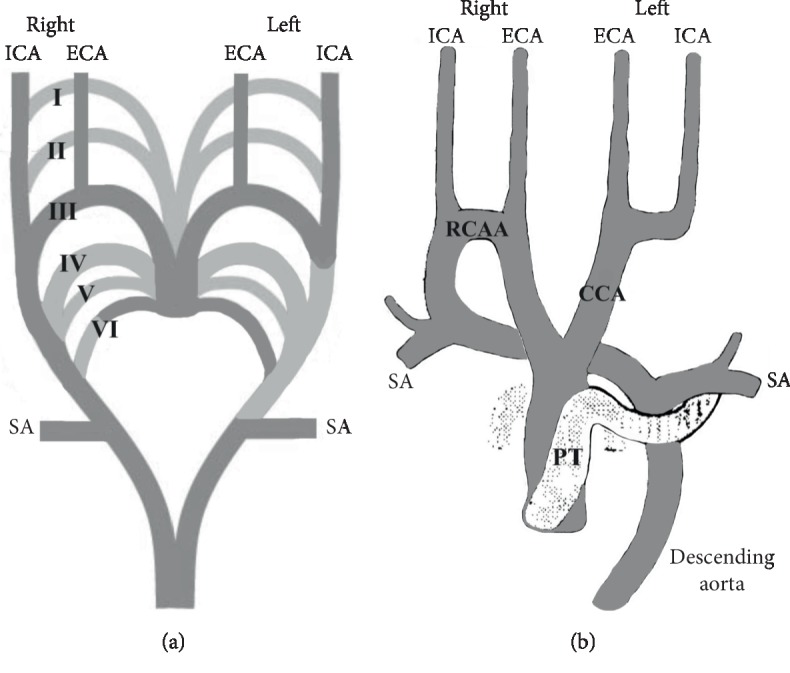
Embryologic basis of the separated origin of the right external carotid (ECA) and internal carotid (ICA) arteries and right-sided cervical aortic arch (RCAA) in modified Myers' scheme [10] (a), and course and branches of the RCAA in modified Beavan's and Fatti's scheme [36] (b). Note: Obliterated and persisted parts are light- and dark gray colored, respectively. I–VI, primitive aortic arches; SA, subclavian artery; CCA, common carotid artery; PT, pulmonary trunk.

The right-sided cervical aortic arch (RCAA) was also found as a vascular source of the right ECA and ICA (Figure 3), where the third PAA persisted to take part in the formation of the RCAA [36]. If this was to happen and the right DC was to persist, a high RCAA would occur with the ECA and ICA arising as separate branches from it. If it is, in fact, derived from the third PAA, then the RCAA will incorporate the third PAA and DC; an incorporation of the third PAA into the RCAA should result in the development of the carotid sinus within it [37].

### 3.3. Aplasia of Bilateral CCA

**Figure 4:**
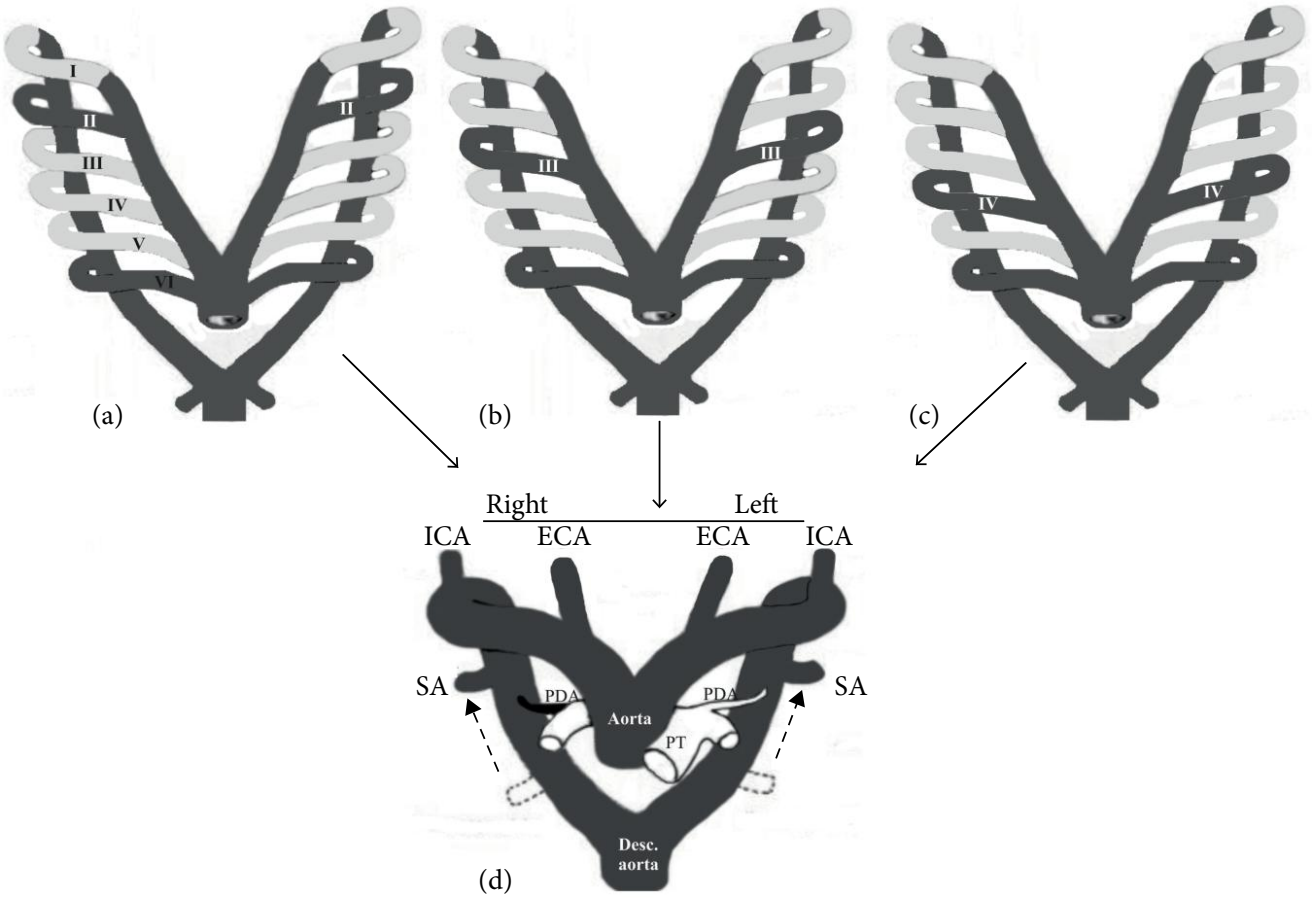
Modified diagrams [39] of the separated origin of both external carotid (ECA) and internal carotid (ICA) arteries in a case of double aortic arch (DAA). Hypothetically, the second (II) aortic arch (a), or the third (III) aortic arch (b), or the fourth (IV) aortic arch (c) can be incorporated in the DAA, which is a vascular source of bilateral ECAs and ICAs. Note: Obliterated and persisted parts are light- and dark gray colored, respectively. SA, subclavian artery; PDA, persistent ductus arteriosus; PT, pulmonary trunk.

Supsupin et al. [38] sketched the separated origin of the both-sides ECA and ICA as the consequence of an obliteration of the third PAA followed by the persistence of the DC on the both sides, while Shuford et al. [39] sketched the interruption of the left embryonic arch between the left SA and the carotid arteries in a hypothetical double aortic arch with an independent origin of the carotid arteries (Figure 4).

## 4. Unilateral Aplasia versus Bilateral Aplasia of the CCA

### 4.1. History

Malacarne in 1784 [40], as cited [14], was the first to report the bilateral CCA aplasia, while Gottschau in 1885 [41], as cited [42], was the first to report the unilateral CCA aplasia. A real angiographic evidence of the CCA aplasia on the left side was reported just in the second half of the 20th century [43].

### 4.2. General Data

We evidenced 87 literature cases, which were discovered and/or quoted in the period from 1784 to the present day in various (14 known) countries. There are 34 cases of the left CCA aplasia, 41 cases of the right CCA aplasia and 6 cases of bilateral CCA aplasia, plus 6 cases of unknown lateralization—the two literature cases cited by Macalister [13], Broman's [44] case, cited by Fife [45], Lie's [30] case cited by Uchino et al. [46], Haughton and Rosenbaum's [47] case cited by Bryan [43], and one Weinberg's [48] case. These cases were found in the patients or cadavers of both gender (37 of female, 24 of male and 26 of unknown sex), between the age of a newborn and 77 (Table 1).

It is very difficult to determine a real incidence of the CCA aplasia. Warschewske and Benndorf [42] have noted that the separate origins of the left ECA and ICA constituted an anomaly with an estimated prevalence of <0.1% in the 11 case reports published in the English-language literature, while Faggioli et al. [49] discovered one case of the left CCA aplasia among 214 patients of Italian population; this incidence amounts to 0.46%. Given that only 3 cases of the CCA aplasia were identified among 17,500 patients of the USA population [18], this incidence places at roughly 0.017%.

### 4.3. Diagnosing

It should be borne in mind that the left ICA or ECA of the aortic origin could be mistaken for a left VA that can arise, as cited [16, 50], directly from the aortic arch in approximately 5% of patients or in 9–11% of human fetuses, respectively. Further, it was also noted that the diagnosing by color-Doppler ultrasound could be difficult since intrathoracic bifurcation of the CCA may simulate a separate origin of the left ECA and ICA [11]; however, using this method as cited [23], the vessel that exhibited a low-resistance flow pattern coincides with the ICA, while the vessel that exhibited a high-resistance flow pattern coincides with the ECA [23].

Other recommended methods by some authors [22, 51] were applied in the detection of the particular beginnings of the ECA and ICA; the first method was a gadolinium-enhanced three dimensional magnetic resonance angiography (3D-MRA) because of its perfect resolution and diagnostic accuracy [22], and the second method was a multidetector computer tomography (MDCT) with the angiographic protocol: imaging acquired and multiplanar 3D reconstruction [51].

### 4.4. Vascular Sources of the Separated ECA and ICA Origin

We presented the various vascular sources of the independent origin of the ECA and ICA.

#### 4.4.1. Normal Aortic Arch (NAA)

**Figure 5:**
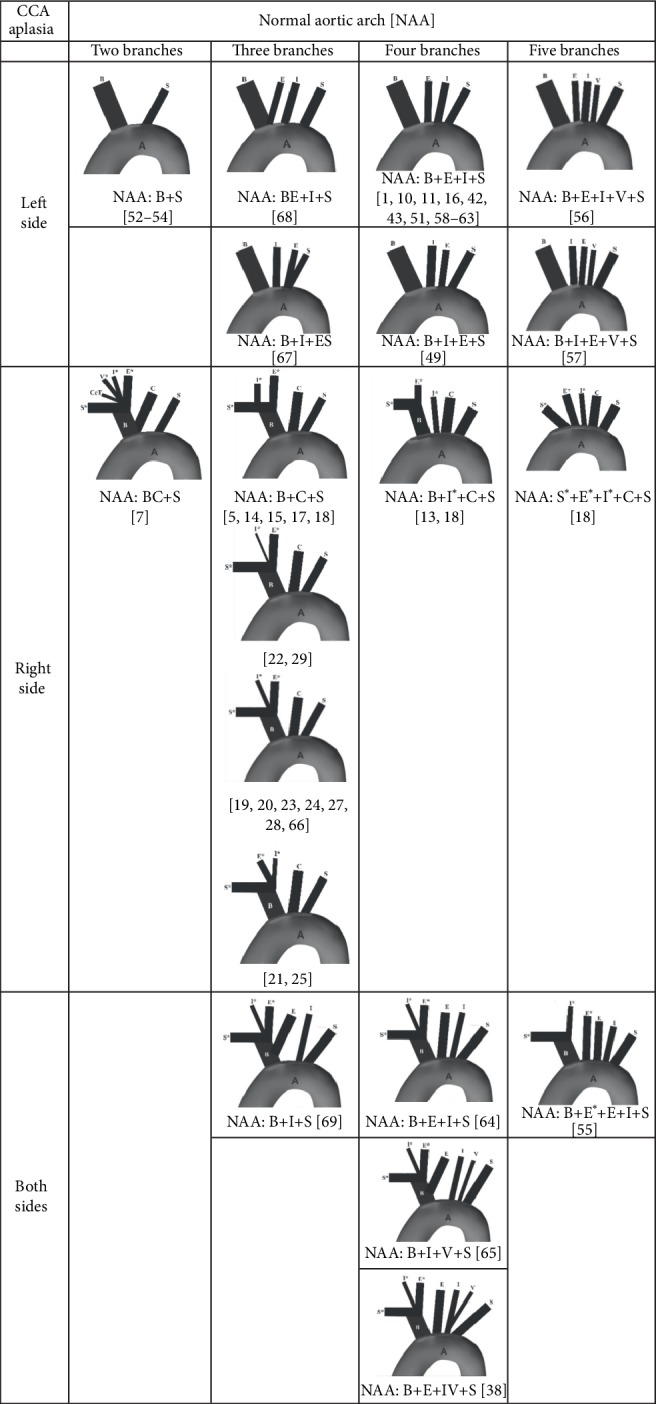
Patterns of the branching of the normal aortic arch in cases of uni- and bilateral aplasia of the common carotid artery (CCA). Note: Kosinski, 1868 [66], cited by Boyd [14]. A, aorta; B, brachiocephalic trunk, S (S^∗^), left (right) subclavian artery; E (E^∗^), left (right) external carotid artery; I (I^∗^), left (right) internal carotid artery; V (V^∗^), left (right) vertebral artery; C, left common carotid artery; CcT, costocervical trunk.

 The ECA and ICA originated particularly from the NAA in 29/34 or 85.29% of the cases on the left, and in 24/41 or 58.53% of the cases on the right side. The patterns of the NAA branching were different in these cases; the number of these branches ranged from two [7, 52–54] to five [18, 55–57]. Hovewer, the NAA with four branches was a more common finding associated with the left CCA aplasia [1, 10, 11, 16, 42, 43, 49, 51, 58–63], than with the right [13, 18, 26] or bilateral CCA aplasia [38, 64, 65]; a pattern of the NAA with three branches was more common in the cases of the right CCA aplasia [5, 14, 15, 17–25, 27–29, 66], than in the cases of the left [67, 68] or bilateral CCA aplasia [69] (Figure 5).

#### 4.4.2. Cervical Aortic Arch

**Figure 6:**
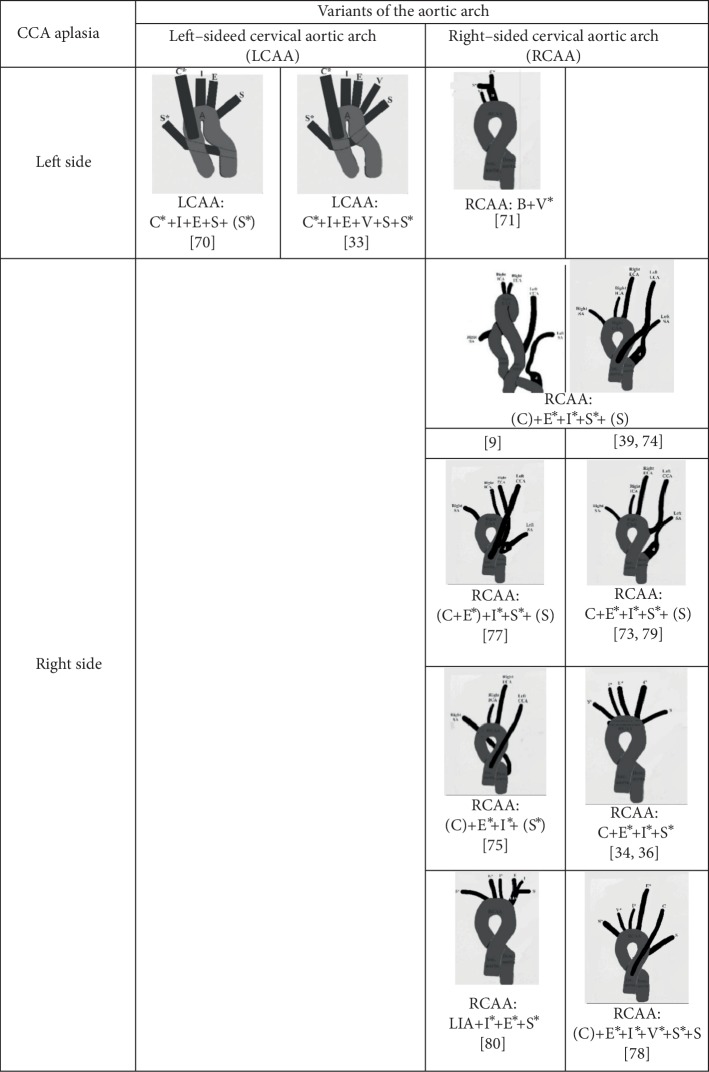
Patterns of the branching of the ascending aorta, left-sided and right-sided cervical aortic arches, and descending aorta in cases of unilateral aplasia of the common carotid artery (CCA). Note: Mullins et al., 1973 [78], cited by Haughton et al. [34]. Capital letter(s) in the small parenthesis indicate the vessel(s) originating from the ascending aorta, while these in the square parenthesis indicate the vessel originating from the descending aorta; other letters indicate the vessels branched from the arch of the aorta. A, aorta; C (C^∗^), left (right) common carotid artery; S (S^∗^), left (right) subclavian artery; E (E^∗^), left (right) external carotid artery; I (I^∗^), left (right) internal carotid artery; V (V^∗^), left (right) vertebral artery; LIA, left innominate artery.

 The separated left ECA and ICA originated from the left-sided cervical aortic arch (LCCA) in 2/34 cases [33, 70] and from the right-sided cervical aortic arch (RCAA) also in 2/34 cases [71, 72]; the right ECA and ICA originated from the RCAA (Figure 6) in 14/41 cases [9, 34, 36, 39, 45, 73–81].

#### 4.4.3. Double Aortic Arch (DAA) and Interrupted Aortic Arch (IAA)

**Figure 7:**
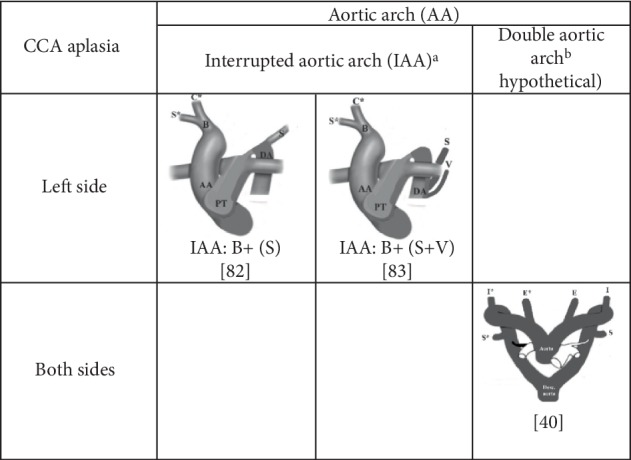
Status and branches of the interrupted (IAA) and double (DAA) aortic arches in cases of the unilateral and bilateral aplasia of the common carotid artery (CCA). ^a^Modified schema of the IAA [82]. ^b^Modified schema of the DAA [39]; Malacarne, 1784 [40], cited by Andrews and Howard [6]. Note: Capital letter(s) in the square parenthesis indicate the vessel(s) originating from the descending aorta; other letters indicate the vessels branched from the arch of the aorta. PT, pulmonary trunk; B, brachiocephalic trunk; S (S^∗^), left (right) subclavian artery; C^∗^, right commmon carotid artery; V, left vertebral artery; DA, descending aorta; I (I^∗^), left (right) internal carotid artery; E(E^∗^), left (right) external carotid artery.

There was one case of the bilateral origin of the ECA and ICA from the DAA [40], as cited [6], while there were two cases of aplasia of all the left carotid vessels in the presence of the IAA [82, 83] (Figure 7).

#### 4.4.4. Brachiocephalic Trunk (BT)

This trunk had a common origin with the left ECA only in one case [68]. However, it was the vascular source of the right ECA in 4/41 cases [14, 15, 17, 18], and the separated right ECA and ICA in 15/41 cases of the right CCA aplasia [5, 7, 19–29, 66, 84]. The BT distributed the left ECA in 2/6 cases [65, 69], and both of the right ECA and ICA in 5/7 cases of the bilateral CCA aplasia [38, 55, 64, 65, 69].

#### 4.4.5. Subclavian Artery (SA)

The right ICA had the SA origin in 4/41 cases [14, 17, 18, 22].

#### 4.4.6. Persistent Proatlantal Intersegmental Artery (PPIA)

A unique case represents the origin of the left ECA and ICA from the ipsilateral PPIA at the level of C III vertebra [54].

### 4.5. Caliber of the ECA and ICA

There were no data about the diameter size of the separated left or right ECA and ICA, except for the data about a “small” caliber or hypoplasia of the left ICA in 3/34 cases [54, 61, 62] or of the right ICA in 3/41 cases [5, 22, 29].

### 4.6. Course of the Separated ECA and ICA

There were no data about the variable branches of the ECA, or the deviation from the normal course of the ECA and/or ICA after their particular origin, except for the tortuosity of the left ICA in 5/34 cases [42, 61, 62, 85, 86] and of the right ICA in 1/41 cases [14].

### 4.7. Summarized Vascular Variations

The associated vascular variations with uni- or bilateral aplasia of the CCA were showed in the Table 2; these were found in the articles by the authors cited in the previous headings, except of Johnson et al. [87].

#### 4.7.1. Collateral Pathways

The collaterals were activated between the muscular branches of the left VA and left ECA [52, 53], or between the left accessory meningeal artery and the inferolateral trunk [62] in a case of the left CCA aplasia.

There were tortuous collaterals along the left side of the spine at the level of C VI–C VII vertebrae in a boy with the right CCA aplasia [74]. In addition, there were anastomoses between the right occipital artery and the right VA, and between the right ascending cervical artery and the thyrocervical trunk in a man with the right CCA aplasia [29].

The collaterals between the both-sides thyrocervical trunks and the right bronchial artery were proved in a girl with bilateral aplasia of the CCA [64].

The subclavian origin of the right ICA provided the ICA contribution to the SA steal syndrome in the presence of the SA stenosis [5]. A complete atresia of the left SA at the origin from the RCAA associated/ together with the particular origin of the right ECA and ICA had as a consequence the collateral supply of the SA [75]. A retrograde blood supply to the left VA and the arteries of the left upper limb was enabled by the right VA in a few cases of the left CCA aplasia [71].

### 4.8. Associated Pathology

We had no complete data for 14/87 cases [10, 20, 40, 41, 66, 72, 76, 78, 85–90]. In relation to the cases with known data, there was no associated pathology in 13/73 cases [1, 11, 16, 18, 23, 26, 28, 43, 49, 51, 59]. However, 21 various pathological states in other (60) cases were as follows: The amaurosis fugax [15], aneurysms of some cerebral arteries [24, 31, 42, 52, 53, 58, 61, 62, 67, 71], arteriovenous fistula [57], the carcinoma of the stomach [14], cerebral infarction [7, 27, 84], cervical spinal fusion [38], a cerebral vascular accident [25], a congenital cardiovascular anomaly [34, 56, 64, 79, 80, 82, 83], myocardial infarction [21], multiple block vertebrae [69], multiple sclerosis [22], Noonan syndrome [62], the occlusion of artery [5, 7, 63], parathyroid adenoma [60], posttraumatic artery dissection [29], pseudoaneurysm [57]; pulmonary embolism [55], the stenosis of artery [5, 7, 15, 22, 25, 70, 75, 84], a subacute frontal intraparenchymal hemorrhage [17], the thrombosis of venous sinuses [68], Turner syndrome [70], and vestibular neuritis [54] (Table 3).

This group also includes five cases of the CCA aplasia [13, 19, 45, 65, 91] that were discovered during routine dissections, but without documented pathoanatomical findings.

## 5. Discussion and Conclusion

An opinion, almost a century old, refers to the statement that a rarity of the separated origins of the ECA and ICA is but an expression of the constancy through which the DC becomes obliterated; actually, this obliteration is one of the most constant features in the varying modifications undergone by the aortic-arch system in higher vertebrates [45].

In general, it can be said that the CCA aplasia is a real rarity. However, the awareness of the possibility of the separate beginnings of the ECA and ICA is important because they are a potential source of confusion on the CTA or MRA, and also on a catheter angiography [63]. The variations of branching of the LCAA or RCAA or NAA, can pose a challenge to the blind catheterization of the supra-aortic vessels, as in the case given by Supsupin et al. [38]. In addition, the beginning of the ECA and ICA from the CAA ensures the screening for 22q11 chromosome deletion [48].

Although a headache was more common among them, it was also an initial symptom in other cases of vascular absence, such as the uni- or bilateral absence of the VA [92], or the bilateral absence of the ICA [93], as well as in many pathological disorders. A suspect pulsatile swelling in the neck caused by the cervical aortic arch, as a reason of/for the discovery of the CCA aplasia [6, 9, 33, 34, 36, 39, 67, 70, 73–75, 77, 81], may not always be related to it, as evidenced by Shepherd et al. [37].

As previously mentioned by Wood et al. [18], the key parameters included the cases explicitly detailing the CCA absence and did not include the cases such as those describing the additional absence of the ICA or ECA, which could indirectly result in the CCA aplasia. We included all the cases of the unilateral absence of all the carotid (common/external/internal) arteries [18, 31, 53, 71]. Summarizing these data we have recorded 87 published and/or cited cases of the CCA aplasia in the last 233 years (1784–2017). This number was increased by four when we took into account the following cases: Nizankowski et al. [94], as cited [32] have only reported the independent aortic origin of the left ECA and ICA as the type XIII without any additional data; Kunishio et al. [53, 95] reported the same case twice; Kobayashi et al. [96] described the bilateral absence of the CCA and ICA, but they did not either mention the status of the ECAs; and Uchino et al. [46] labeled the separated right ICA and ECA origin from “the BT” and not from the right CCA, although the right SA had the independent origin from the NAA.

We can support an earlier statement that the CCA aplasia had no significant left–right side preferences [7, 43]; however, if only the cases of the known sex were taken into account, the cases of female gender were more common. The CCA aplasia was proven in one newborn [82], and in one neonate [56], but also in people at the age of 70 and above [7, 18, 21, 25, 53, 60, 63].

We summarized 16 different vascular variants; the aplasia of the BT, or the origin of the separated right ECA and the right ICA from the BT or the RCAA were more common among them. A case of the separated origin of the left ECA and ICA from the PPIA was a unique case [54], while the aortic arch either in the form of the NAA or LCAA or RCAA or DAA was a vascular source of the separated ECA and ICA in most of the cases. Only the left ECA and ICA, although rarely, originated from the LCAA [33, 70], or RCAA [71, 72]; the right ECA and ICA originated from the RCAA in one third of the cases.

There was no associated pathology in 17.80% of cases; however, there were 21 different pathological states in other cases, and among them the aneurysms of some cerebral arteries in 13.69% [24, 31, 42, 52, 53, 58, 61, 62, 67, 71].

The authors have presented the morphofunctional status of blood vessels and stated the clinical importance in the cases of the absence of the VA [92], or the bilateral ICA [93], and the CCA in this manuscript; the next task will be a comparison of the cases with the left and the ones with the right ICA absence.

## Figures and Tables

**Table 1:** Distribution of cases of the common carotid artery (CCA) aplasia according to age and gender.

Side of the CCA aplasia	New-born (few hours after birth)	Neo-nate (≤28 days)	Suck-ling (≤12 months)	Tot (≤3 years)	Pre- school age (≤5)	School age (6–12)	Adolescents (13–18)	19–29	30–39	40–49	50–59	60–69	70–79	*U*	∑
Bilateral				F			M	F+M						M+U	6 (2F+3M+U)
Left	M	U					F	F+M	3F	6F+3M	F	4F	3M	9U	34 (16F+8M+10U)
Right				U	M	5F+4M	F	M	F+2M	F	4F+2 M	3F+2 M	3F+M	(F+8U)	41 (19F+13M+9U)
*U* ^∗^														6	6 (6U)
∑	1 (M)	1 (U)		2 (F+U)	1 (M)	9 (5F+4M)	3 (2F+M)	5 (2F+3M)	6 (4F+2M)	10 (7F+3M)	7 (5F+2M)	9 (7F+2M)	7 (3F+4M)	26 (F+M+24U)	87 (37F+24M+26U)

U: unknown; F: female; M: male.

**Table 2:** Summarized vascular variants^a^ in cases of the common carotid artery (CCA) aplasia.

No	Kind of vascular variant	Target artery (side/mode of the origin)	CCA absence		
			Left side	Right side	Bilateral
			References		
1	Accessory vessel	Lateral IThA		[45]	
2	Additional absence	Left ACA	[61]		
		Left OPhA	[52]		
		Right ACA		[22]	
		BT		[9, 18, 34, 36, 39, 45, 73–75, 77–79, 81]	[80]
		Left ECA	[18, 52, 53, 71, 83]		
		Left ICA	[18, 52, 53, 71, 83]		
		Left SA	[71]	[74, 75]	
		Left VA	[71]		
		Right ECA		[31, 74]	
		Right ICA		[31, 74]	
		Right TT		[19]	
3	Arterial loop	Left ICA	[16]		
		Right ECA/ICA			[64]
4	Diverticulum at the origin	Left SA		[9, 39, 45, 73–75, 77, 79]	
5	Ectasia	Left PCoA	[52, 53]		
		Left SA	[3]		
		Left VA	[54]	[29]	
		Right VA		[29]	
6	Elongation	BT		[19, 84]	
7	Hypoplasia	Left A1	[42, 58]		
		Right A1		[24]	
		Left ECA	[54]		
		Left ICA	[54, 61, 62]		
		Right ICA		[5, 22, 29]	
		Right VA	[58]		
8	Low bifurcation	Right CCA	[1, 68]		
9	Persistence of the primitive CVBA	Left PPIA (type 2)	[54, 59]		
		PPIA	[41]		
		Left PTAV	[58]	[29]	
10	Persistence of the primitive vein	Left SVC		[45]	
11	Short trunk	BT	[6]		
12	Tortuosity	Anastomotic vessels		[29]	
		BT		[84]	
		Left ICA	[42, 61, 62, 85, 86]		
		Left SA	[33]		
		RCAA		[79]	
		Right ICA		[14]	
		Right SA	[83]		
13	Unusual vessel	DAA			[40]
		LCAA	[33, 70]		
		LIA		[80]	
		RCAA	[71, 72]	[9, 34, 36, 39, 45, 73–81]	
14	Variable course	Descending aorta	[33]		
		Left SA		[34, 73]	
		Right VA		[14]	
15	Variable mode of the origin	Fetal origin of the right PCoA	[58]		
		Common origin of the BT and left CCA		[7]	
		Common origin of the BT and left ECA	[68]		
		Common origin of the left ICA and left SA	[67]		
		Common origin of the left ICA and left VA	[87]		[38]
		Distal origin of the right VA		[17]	
		High origin of the right SA			[64]
		Origin of the right ICA proximally to the right ECA		[21, 25]	
16	Variable vascular source: branch(es)	Ascending aorta: left CCA		[74, 75, 77, 81]	
		Ascending aorta: right ECA		[77]	
		BT: left ECA			[65, 69]
		BT: left VA	[10]		
		BT: right CcT		[7]	
		BT: right ECA		[5, 14, 15, 17, 18]	
		BT: right ECA and right ICA		[7, 20–29, 66, 84]	[38, 65, 69]
		BT: right ICA			[55]
		BT: right VA		[7]	
		Descending aorta: left VA	[83]		
		Descending aorta: left SA	[82, 83]	[9, 39, 75]	
		Descending aorta: right SA		[75]	
		DAA: left and right ECAs and ICAs			[40]
		LIA: left CCA and SA /		[80]	
		Left MCA: left OPhA	[53]		
		Left PCoA: left MCA	[53]		
		Left PPIA: left ECA and ICA	[54]		
		LCAA: left ECA and left ICA	[33, 70]		
		NAA: left ECA and ICA			[55]
		NAA: left ICA			[38, 65, 69]
		NAA: left VA		[26]	[38, 65]
		NAA: right ECA			[55]
		NAA: right ECA and right ICA		[18]	
		NAA: right ICA		[13, 18]	
		NAA: right VA	[71]		
		RCAA: right ECA and right ICA	[71, 72]	[34, 36, 45, 73, 75–81]	
		RCAA: right SA		[9, 34, 36, 39, 73, 74, 77–80]	
		RCAA: right VA		[78]	
		Right ECA: right IThA		[19]	
		Right ICA: right APhA		[19]	
		Right SA: right ICA		[5, 14, 15, 17, 22]	

^a^Vascular variants are only alphabeticaly listed and do not indicate the number of cases, because they were multiple in some of the cases.

Note: Some references are cited as follow: Malacarne [40], cited by Andrews and Howard, [6]; Gottschau [41], Smirnov [85], and Schmeidel [86], cited by Bryan et al. [43]; Kosinski [66], cited by Boyd [14]; Chang et al. [76], cited by Shuford et al. [39]; and Johnson et al. [87], cited by Dahn et al. [16].

IThA internal thoracic artery, ACA anterior cerebral artery, OPhA ophthalmic artery, BT brachiocephalic trunk, ECA external carotid artery, ICA internal carotid artery, SA subclavian artery, VA vertebral artery, TT thyrocervical trunk, PCoA posterior communicating artery, Al pre-communicating part of the ACA, PPIA persistent proatlantal intersegmental artery, PTAV persistent trigeminal artery variant, SVC superior vena cava, RCAA right-sided cervical aortic arch, DAA double aortic arch, LCAA left-sided cervical aortic arch, LIA left innominate artery, CcT costocervical trunk, MCA middle cerebral artery, NAA normal aortic arch, APhA ascending pharyngeal artery.

**Table 3:** Presence and absence of pathoanatomical states in 87 cases with the common carotid artery (CCA) aplasia.

Pathological diagnoses or findings^a^				Left CCA absence	Right CCA absence	Bilateral CCA absence
				References		
Pathology *Autopsy*	Amaurosis fugax				[15]	
	Aneurysm	ACoA		[52, 53]	[31]	
		BA		[52]		
		ICA (left)		[61, 62, 67]		
		ICA (right)		[42, 58, 71]		
		A1–A2 (left)			[24]	
		MCA (left)		[53]		
		SCA (left)		[58]		
	Arteriovenous fistula			[57]		
	Carcinoma of stomach				[14]	
	Cerebral infarction				[7, 27, 84]	
	Cerebral vascular accident				[25]	
	Cervical spinal fusion					[38]
	Congenital cardiovascular disease			[56, 82, 83]	[34, 79]	[55, 64, 80]
	Myocardial infarction				[21]	
	Multiple block vertebrae					[69]
	Multiple sclerosis				[22]	
	Noonan syndrome			[62]		
	Occlusion of artery	ICA (left)			[5, 7]	
		SA (right)			[5]	
		VA (right)		[63]		
	Parathyroid adenoma			[60]		
	Posttraumatic artery dissection	ICA (right)			[29]	
	Pseudoaneurysm	MA (left)		[57]		
		FA (left)		[57]		
	Pulmonary embolism					[55]
	Stenosis of artery	ICA (left)			[7, 15]	
		ICA (right)			[5, 7, 15, 22, 25, 84]	
		SA (right)		[70]		
		SA (left)			[75]	
	Subacute frontal intraparenchymal hemorrhage				[17]	
	Thrombosis	TS (right)		[68]		
		SSS		[68]		
	Turner syndrome			[70]		
	Vestibular neuritis			[54]		
					[13, 19, 45, 91]	[65]

No pathology				[1, 11, 16, 43, 49, 51, 59]	[18, 23, 26, 28]	

No data				[10, 41, 72, 85–89]	[20, 66, 76, 78, 90]	[40]

^a^Pathological disorders and findings only alphabetically listed and do not indicate the number of cases, because they were multiple in some of the cases.

Note: Some references are cited as follow: Quain [91], cited by Fife [45]; Malacarne [40], cited by Andrews and Howard, [6]; Gottschau [41], Von Angermayer [88], Smirnov [85], and Schmeidel [86], cited by Bryan et al. [43]; Kosinski [66], cited by Boyd [14]; Merkel and Bonnet [89], cited By Warschewske and Benndorf [42]; Mullins et al. [78], cited by Haughton et al. [34]; Chang et al. [76], cited by Shuford et al. [39]; and Johnson et al. [87], cited by Dahn et al. [16].

ACoA anterior communicating artery, BA basilar artery, ICA internal carotid artery, A1–A2 junction of the pre- and postcommunicating parts of the anterior cerebral artery, MCA middle cerebral artery, SCA superior cerebellar artery, SA subclavian artery, VA vertebral artery, MA maxillary artery, FA facial artery, TS transverse sinus, SSS superior sagittal sinus.
